# The Chinese Medicinal Formulation Guzhi Zengsheng Zhitongwan Modulates Chondrocyte Structure, Dynamics, and Metabolism by Controlling Multiple Functional Proteins

**DOI:** 10.1155/2018/9847286

**Published:** 2018-11-22

**Authors:** Baojin Yao, Bocheng Lu, Mei Zhang, Hongwei Gao, Xiangyang Leng, Daqing Zhao

**Affiliations:** ^1^Jilin Ginseng Academy, Changchun University of Chinese Medicine, Changchun, Jilin 130117, China; ^2^The Affiliated Hospital of Changchun University of Chinese Medicine, Changchun, Jilin 130117, China; ^3^Innovation Practice Center, Changchun University of Chinese Medicine, Changchun, Jilin 130117, China

## Abstract

Traditional Chinese medicine is one of the oldest medical systems in the world and has its unique principles and theories in the prevention and treatment of human diseases, which are achieved through the interactions of different types of materia medica in the form of Chinese medicinal formulations. GZZSZTW, a classical and effective Chinese medicinal formulation, was designed and created by professor Bailing Liu who is the only national medical master professor in the clinical research field of traditional Chinese medicine and skeletal diseases. GZZSZTW has been widely used in clinical settings for several decades for the treatment of joint diseases. However, the underlying molecular mechanisms are still largely unknown. In the present study, we performed quantitative proteomic analysis to investigate the effects of GZZSZTW on mouse primary chondrocytes using state-of-the-art iTRAQ technology. We demonstrated that the Chinese medicinal formulation GZZSZTW modulates chondrocyte structure, dynamics, and metabolism by controlling multiple functional proteins that are involved in the cellular processes of DNA replication and transcription, protein synthesis and degradation, cytoskeleton dynamics, and signal transduction. Thus, this study has expanded the current knowledge of the molecular mechanism of GZZSZTW treatment on chondrocytes. It has also shed new light on possible strategies to further prevent and treat cartilage-related diseases using traditional Chinese medicinal formulations.

## 1. Introduction

Chinese materia medica (CMM), the main form of traditional Chinese medicine (TCM), is widely used by a large, global population. The World Health Organization (WHO) also encourages member countries to include traditional medicine and materia medica in their primary healthcare systems. Therefore, TCM and CMM have been used by people and communities all over the world [1]. TCM is one of the oldest medical systems in the world and has unique principles and theories in the prevention and treatment of human diseases. The effects of TCM are achieved through the interactions of different types of materia medica, which are combined to make Chinese medicinal formulations. These formulations have been used and tested in humans for many centuries, from ancient times to today. Furthermore, tens of thousands of clinical reports have been published up till now [2].

Osteoarthritis (OA) is the most common joint disease characterized by articular cartilage degeneration accompanied by inflammation and osteophyte formation. There are some remedies for OA to relieve pain, such as analgesics, nonsteroidal anti-inflammatory drugs (NSAIDs), or joint replacement surgery. However, none of these treatments are sufficient to prevent the progression of OA. This is due to the poor repair and regeneration potentials of chondrocytes, the only cell type in cartilage, which controls cartilage structure and function [3]. Moreover, OA is a leading cause of disability worldwide, and, currently, there is no effective treatment for OA patients [4].

Guzhi Zengsheng Zhitongwan (GZZSZTW) is an effective Chinese medicinal formulation and has been used for the treatment of OA patients in the Affiliated Hospital of Changchun University of Chinese Medicine for several decades. This formula is orally administered in the form of pills prepared in boiled water, which are only used in the hospital's internal preparation center to treat osteoarthritis patients. However, our knowledge of the regulatory role of GZZSZTW on primary chondrocytes and the underlying mechanism remains largely unknown. In the present study, we performed quantitative proteomic analysis to investigate the effects of GZZSZTW on mouse primary chondrocytes using state-of-the-art iTRAQ technology. We demonstrated that the Chinese medicinal formulation GZZSZTW modulates chondrocyte structure, dynamics, and metabolism by controlling multiple functional proteins that were involved in the cellular processes of DNA replication and transcription, protein synthesis and degradation, cytoskeleton dynamics, and signal transduction.

## 2. Materials and Methods

### 2.1. Preparation of the GZZSZTW Aqueous Extract

GZZSZTW medicinal formulation, which consisted of* Rehmannia glutinosa *(Gaertn.) DC.,* Spatholobus suberectus *Dunn,* Epimedium brevicornu Maxim *(K.S.Hao),* Raphanus sativus *L. (Hook. f. & T. Anderson) (baked),* Drynaria fortunei *(Kunze ex Mett.) J.Sm. (baked),* Cynomorium coccineum *subsp.* songaricum *(Rupr.) (J.Léonard), and* Cibotium barometz *(L.) (J.Sm), was provided by the Affiliated Hospital of Changchun University of Chinese Medicine (Changchun, China). The mixture of GZZSZTW was immersed in distilled water for 30 min. Subsequently, the mixture was decocted with a 10-fold volume of distilled water by a refluxing method and was then filtered through a 0.45 *μ*m Hollow Fiber Cartridge (GE Healthcare, USA). The filtrate was freeze-dried using a Heto PowerDry LL3000 Freeze Dryer (Thermo, USA) and stored at -80°C [5].

### 2.2. Primary Chondrocytes Isolation and Treatment

Animal experiments were approved by the Ethical Committee for Animal Research of Changchun University of Chinese Medicine. Primary chondrocytes were isolated and cultured as previously described [6]. Briefly, rib cages of C57BL/6J neonatal mice were dissected under sterile conditions and the cartilage was carefully separated and digested with 3 mg/ml collagenase D for 45 min and then overnight with 0.5 mg/ml collagenase D (Sigma, USA). The cells released from the tissue were centrifuged and suspended in DMEM/F12 medium (Thermo, USA) containing 5% FCS (Thermo, USA) and 1% penicillin/streptomycin (Sigma, USA). Primary chondrocytes were seeded into 6-well culture dishes at a density of 1 × 10^5^ cells/well (2 ml per well) and cultured at 37°C in an incubator (Thermo, USA) containing 5% CO_2_ for 4 h. Then, the culture medium was discarded, and each well was completely rinsed with fresh culture medium. The chondrocytes were treated with either 0.8 mg/ml GZZSZTW dissolved in culture medium or plain culture medium for 24 h [5].

### 2.3. Preparation of Protein Extracts

The culture medium was discarded, and each well was completely rinsed with ice-cold phosphate-buffered saline (pH 7.4, 137 mM NaCl, 2.7 mM KCl, 4.3 mM Na_2_HPO_4_, and 1.4 mM KH_2_PO_4_). Primary chondrocytes were suspended in the lysis buffer (7 M Urea, 2 M Thiourea, 4% CHAPS, 40 mM Tris–HCl (pH 8.5), 1 mM PMSF, and 2 mM EDTA) and sonicated for 30 min on ice. Then, 10 mM DTT (Sigma, USA) was added and incubated at 56°C for 1 h followed by the addition of 55 mM IAM (Sigma, USA) and incubation in the darkroom at room temperature for 45 min. The proteins were precipitated with ice-cold acetone (1:5, v/v) at -20°C for 2 h. After centrifugation at 4°C for 15 min, the pellet was dissolved in 0.5 M TEAB (Sigma, USA) and sonicated for 20 min on ice. The supernatant was obtained by centrifuging again and protein concentration was determined using a Bradford protein assay kit (Bio-Rad, USA) [7].

### 2.4. Peptide Labeling and Strong Cation Exchange Fractionation

A total of 100 *μ*g of protein per sample was used for iTRAQ labeling. Protein samples were digested with trypsin (Sigma, USA) with a mass ratio of 20:1 at 37°C for 12 h. The digested proteins were freeze-dried, reconstituted in 0.5 M TEAB (Sigma, USA), and labeled using the iTRAQ Reagent 8 Plex One Assay Kit (AB Sciex, USA) according to the manufacturer's instructions [8]. The untreated blank group was labeled with iTRAQ tag 114 and the GZZSZTW group was labeled with tag 115. The labeled peptide mixtures from the two groups were then equally mixed and dried using a speed-vacuum centrifuge (Thermo, USA). The dried labeled peptides were dissolved in 4 mL of strong cation exchange (SCX) buffer (25 mM NaH_2_PO_4_ in 25% acetonitrile, pH 2.7) and fractionated using an Ultremex strong cation exchange column (Phenomenex, USA) on the LC-20AB high-performance liquid chromatography platform (Shimadzu, Japan) [9].

### 2.5. High-Performance Liquid Chromatography and Mass Spectrometry Analysis

The fractions were further identified and quantified by ultra-high-performance liquid chromatography using a nanoACQuity (Waters, USA) coupled with a Triple TOF 5600 tandem mass spectrometer (AB SCIEX, USA) and subsequently analyzed according to previously described methods [10]. Briefly, online trapping and desalting were performed using microfluidic traps and nanofluidic columns packed with Symmetry C18 (Waters, USA) and analytical separation was carried out using nanofluidic columns packed with BEH130 C18 (Waters, USA). Data were acquired on a Triple TOF 5600 platform using an ion spray voltage of 2.5 kV, a curtain gas of 30 psi, a nebulizer gas of 15 psi, and an interface heater temperature of 150°C. The MS was operated with a resolving power of greater than or equal to 30,000 FWHM for the TOF-MS scans. For information-dependent acquisition, the survey scans were acquired at 250 ms, and as many as 30 product ion scans were collected when exceeding a threshold of 120 counts per second and with a 2+ to 5+ charge-state. The Q2 transmission window was set to 100 Da for 100%. A sweeping collision energy setting of 35 ± 5 eV coupled with the iTRAQ adjusted rolling collision energy was applied to all the precursor ions for collision-induced dissociation. The dynamic exclusion was set for 1/2 of the peak width, and then, the precursor was refreshed off the exclusion list.

### 2.6. Data Analysis

The collected raw data were processed using the Proteome Discoverer software (Thermo, USA). The Mascot search engine (Matrix Sciences, UK) was used to search against the NCBI nonredundant protein (https://www.ncbi.nlm.nih.gov/refseq/) and UniProt databases (http://www.uniprot.org/) [11]. The search results were integrated by the IPeak program [12]. The protein quantification was performed using the IQuant software [13]. The following criteria were used for screening differentially expressed proteins: fold change ≥1.5 or ≤0.67 and *p* value ≤0.05 [14]. Functional annotations were further conducted by searching these proteins against the KEGG (http://www.genome.jp/kegg/pathway.html), eggNOG (http://eggnogdb.embl.de/), and WoLF PSORT databases (https://wolfpsort.hgc.jp/) [15].

### 2.7. Parallel Reaction Monitoring Validation

The parallel reaction monitoring (PRM) assays were used to further validate the expression levels of differentially expressed proteins as previously described [16]. Briefly, SpectroDive software (version 8.0, Biognosys AG, Zurich, Switzerland) was used to generate the spectral library, and the target candidates were selected from the Data Dependent Acquisition (DDA) proteomic runs that were acquired using a Q-EXACTIVE instrument. Data analysis was performed using default settings with minor modifications. Protein fold changes were estimated using Student's* t*-test and the cutoff was 0.05.

## 3. Results

### 3.1. Protein Identification

**Figure 1:**
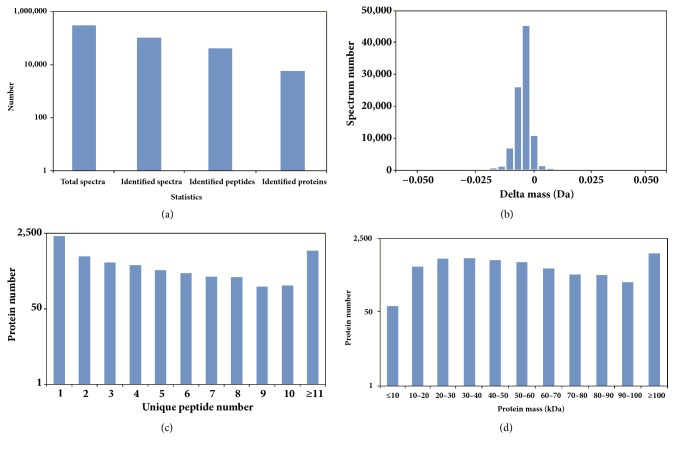
Statistics of protein identification. (a) Numbers of spectra, peptides, and proteins that were identified by iTRAQ identification. (b) The relationship between spectra number and delta mass. (c) Number of unique peptides that match associated proteins. (d) Molecular weight distribution of the proteins that were identified from the iTRAQ analysis.

A total of 312,285 spectra were detected by iTRAQ and 106,325 were identified. Additionally, 41,555 peptides matched to these spectra were identified. In total, based on these identified peptides, 6,026 proteins were identified as shown in Figure 1(a). The newly identified peptides were detected with a delta mass between -0.025 and +0.025 Da (Figure 1(b)). A majority of the identified proteins consisted of at least two unique peptides (Figure 1(c)). The protein masses of more than half of these proteins ranged from 10 to 70 kDa, though their molecular weights covered a wide range (Figure 1(d)).

### 3.2. Functional Annotation of the Differentially Expressed Proteins

We identified a total of 84 differentially expressed proteins according to the iTRAQ analysis. Among these proteins, the expression levels of 67 proteins were significantly increased and 17 proteins were significantly decreased after GZZSZTW treatment. We first performed KEGG pathway enrichment analysis. These differentially expressed proteins were mapped to 25 KEGG pathways, as shown in Table 1. The top eight enriched pathways, according to the numbers of mapped proteins, included translation (12), transport and catabolism (6), signal transduction (6), immune system (5), infectious diseases (specifically viral) (4), cell growth and death (3), cancers (overview) (3), and endocrine system (3).

We then analyzed the subcellular localization of these differentially expressed proteins. As shown in Table 2, the majority of these differentially expressed proteins were distributed in the nucleus (35), cytoplasm (18), and extracellular space (14).

We further analyzed the functions of these differentially expressed proteins based on eggNOG classification. As shown in Table 3, the majority of these differentially expressed proteins were classified into the following functional categories: cytoskeleton (17), posttranslational modification/protein turnover/chaperones (14), signal transduction mechanisms (11), chromatin structure and dynamics (11), translation/ribosomal structure and biogenesis (10), transcription (8), extracellular structures (7), or intracellular trafficking/secretion and vesicular transport (5). To fully explore the mechanisms by which GZZSZTW regulates chondrocyte protein expression, we further dissected the protein expression patterns based on the eggNOG classification in detail.

The 17 differentially expressed proteins that were classified under the category of cytoskeleton, as shown in Table 4, were significantly increased after GZZSZTW treatment; among these were several subtypes of keratin types I and II (Krt79, Krt17, Krt15, Krt42, Krt16, Krt10, Krt5, Krt6a, Krt14, Krt1, and Krt2), tubulin alpha-1B chain (Tuba1b), MARCKS-related protein (Marcksl1), desmoplakin (Dsp), septin-7 (Sept7), tropomodulin-2 (Tmod2), and gamma-synuclein (Sncg).

Among the 14 differentially expressed proteins from the category of posttranslational modification, protein turnover, and chaperones, as shown in Table 5, the levels of 8 proteins were significantly increased after GZZSZTW treatment: pregnancy zone protein (Pzp), MARCKS-related protein (Marcksl1), ovostatin homolog (Ovos), tuberoinfundibular peptide of 39 residues (Pth2), calpastatin (Cast), UBX domain-containing protein 4 (Ubxn4), matrix Gla protein (Mgp), and gamma-synuclein (Sncg). The levels of 6 proteins were significantly decreased under GZZSZTW treatment: alpha-fetoprotein (Afp), lactotransferrin (Ltf), serum albumin (Alb), chymotrypsinogen B (Ctrb1), alpha-2-HS-glycoprotein (Ahsg), and kallikrein 1-related peptidase b1 (Klk1b1).

A majority of the 11 differentially expressed proteins from the category of signal transduction mechanisms, as shown in Table 6, were significantly increased after GZZSZTW treatment, including granulocyte colony-stimulating factor receptor (Csf3r), death-associated protein 1 (Dap), keratin type II cytoskeletal 79 (Krt79), MARCKS-related protein (Marcksl1), PH domain leucine-rich repeat-containing protein phosphatase 2 (Phlpp2), endophilin-B1 (Sh3glb1), tuberoinfundibular peptide of 39 residues (Pth2), craniofacial development protein 1 (Cfdp1), a-kinase anchor protein 8 (Akap8), and disabled homolog 2 (Dab2).

A majority of the 11 differentially expressed proteins from the category of chromatin structure and dynamics, as shown in Table 7, were significantly increased after GZZSZTW treatment; among them were parathymosin (Ptms), histone H1.5 (Hist1h1b), methyl-CpG-binding protein 2 (Mecp2), high mobility group protein HMG-I/HMG-Y (Hmga1), histone H1.3 (Hist1h1d), histone H1.4 (Hist1h1e), glyceraldehyde-3-phosphate dehydrogenase (Gapdh), histone H1.1 (Hist1h1a), histone H2A.V (H2afv), and a-kinase anchor protein 8 (Akap8).

A majority of the 10 differentially expressed proteins that were classified under the category of translation, ribosomal structure, and biogenesis were significantly increased after GZZSZTW treatment, as shown in Table 8; among them were subtypes of 40S and 60S ribosomal proteins (Rps19, Rps27l, Rpl34, Rpl37, Rps28, Rps23, Rpl35, and Rpl36) and Eukaryotic translation initiation factor 1A, X-chromosomal (Eif1ax).

The details of the differentially expressed proteins in the categories of transcription, extracellular structures and intracellular trafficking/secretion/vesicular transport, RNA processing and modification, amino acid transport and metabolism, inorganic ion transport and metabolism, carbohydrate transport and metabolism, replication/recombination and repair, cell cycle control/cell division/chromosome partitioning, and energy production/conversion are described in Table 9.

### 3.3. Parallel Reaction Monitoring Validation

Differentially expressed proteins identified by iTRAQ were further validated by a parallel reaction monitoring (PRM) assay. Twelve differentially expressed proteins, Krt42, Krt5, Krt16, Mgp, Pzp, Rpl35, Rps23, Cfdp1, Ptms, Hist1h1b, Mecp2, and Hist1h1a, were randomly selected for PRM analysis. As shown in Table 10, the fold changes were in agreement with the findings of the iTRAQ analysis with a* p* value ≤0.05.

## 4. Discussion

Currently, there is still no effective western medication for OA. The medications that are used clinically can only help to relieve pain and have adverse side effects including cardiovascular, renal, and gastrointestinal complications; hepatotoxicity and cognitive impairment; and injuries [17]. Recently, Chen and colleagues conducted a meta-analysis and demonstrated that traditional Chinese medicinal formulations were safer and more effective for reducing pain and improving overall physical performance and wellness in treatments of knee OA, and they had a lower risk of adverse events than standard western medication treatments [18]. Therefore, it is time for us to consider using traditional Chinese medications for the treatment of OA, since most of the TCM formulations have been used for many centuries and have been investigated in long-term clinical trials in human beings. The most important mission for us is to elucidate their underlying mechanisms in the prevention and treatment of human diseases so that TCM will be accepted and used all over the world.

GZZSZTW, a classical and effective formulation, is designed and created by professor Bailing Liu who is the only national medical master professor in the clinical research field of TCM and skeletal diseases. For several decades, it has been widely used in the Affiliated Hospital of Changchun University of Chinese Medicine to treat joint diseases. However, the underlying molecular mechanisms are still largely unknown. In the present study, we performed a quantitative proteomic analysis to investigate the effects of GZZSZTW on mouse primary chondrocytes using state-of-the-art iTRAQ technology. We aimed to demonstrate the direct effects of GZZSZTW on chondrocytes and further dissect the regulating factors and related pathways.

We first analyzed the differentially expressed proteins that mapped to KEGG pathways. We demonstrated that GZZSZTW treatment significantly changed the expression levels of multiple proteins involved in 25 KEGG pathways that play important roles in regulating multiple biological processes in chondrocytes, such as cellular processes, environmental information processing, genetic information processing, human diseases, metabolism, and organismal systems. The pathways of cell growth and death, cell motility, cellular community-eukaryotes, transport, and catabolism were classified into cellular processes. The pathways of membrane transport, signal transduction, signaling molecules, and interaction were classified into environmental information processing. The pathways of transcription and translation were classified into genetic information processing. The pathways of cancers, cardiovascular diseases, drug resistance, immune diseases, infectious diseases, neurodegenerative diseases, and substance dependence were classified into the regulation of human diseases. The pathways of carbohydrate metabolism and global and overview maps were classified into metabolism processes. The pathways of digestive, endocrine, excretory, immune, and sensory systems were classified into the regulation of organismal systems. These results suggest that GZZSZTW treatment significantly affects protein synthesis and changes the expression levels of functional proteins that participate in chondrocyte growth, metabolism, signaling transduction, and the regulation of disease.

Consistent with the results discussed above, the majority of the differentially expressed proteins were localized in the nucleus, cytoplasm, and extracellular space, which are the crucial places for gene transcription and regulation, signaling transduction and extracellular matrix secretion, and maintenance in chondrocytes. Unlike other tissues, cartilage is a resilient, smooth, and highly specialized connective tissue. Chondrocytes are the only type of cell in cartilage and they produce a large amount of extracellular matrices to regulate chondrocyte metabolism and function [19].

These differentially expressed proteins were further annotated based on BLAST searches against the eggNOG database that provides orthologous groups of proteins at different taxonomic levels with integrated and summarized functional annotations [20]. Our results demonstrated that the majority of these differentially expressed proteins were classified into the categories of cytoskeleton, posttranslational modification/protein turnover/chaperones, signal transduction mechanisms, chromatin structure and dynamics/translation, and ribosomal structure and biogenesis. These results were consistent with our previous KEGG pathway and subcellular localization analysis. Therefore, we further analyzed these differentially expressed proteins in detail according to the eggNOG annotation.

According to the eggNOG annotation, there were 17 differentially expressed proteins that were from the cytoskeleton category. Their expression levels in primary chondrocytes were significantly increased after GZZSZTW treatment. These proteins mainly consisted of type I and type II keratins including Krt79, Krt17, Krt15, Krt42, Krt16, Krt10, Krt5, Krt6a, Krt14, Krt1, and Krt2. Keratins are intermediate filament-forming proteins that provide mechanical support for the cytoskeleton of many cell types, such as chondrocytes. Type I and type II keratins play crucial roles in diverse cellular processes including cell shape adaptation, division, migration and extracellular matrix assembly, and secretion [21, 22]. Our results showed that the expression levels of several other cytoskeleton proteins including Tuba1b, Marcksl1, Dsp, Sept7, Tmod2, and Sncg were also increased after GZZSZTW treatment. Tuba1b is one of the major components of microtubules, which are one of the three principal types of protein filament that comprise the cell cytoskeleton [23]. Marcksl1 is a member of the Marcks family of proteins that play a pivotal role in actin cytoskeletal organization, the protein kinase C signaling pathway, and the calmodulin signaling pathway [24]. Dsp is a critical component of desmosome structures, which are adhesive junctions at adjacent cell contacts that maintain the structural integrity of tissues that undergo mechanical stress by anchoring the keratin cytoskeleton to the desmosomal plaque [25]. In addition, Sept7, Tmod2, and Sncg are three crucial regulators of cytoskeleton dynamics that are involved in microtubule regulation, keratin network, and actin filament assembly and organization [26–28]. Taken together, these results suggest that GZZSZTW directly targets the cytoskeletal networks of primary chondrocytes. Therefore, GZZSZTW might change cytoskeleton dynamics and enable chondrocytes to fulfill their biological functions, thus providing an effective treatment for OA.

According to the eggNOG annotation, there were 14 differentially expressed proteins that were classified into the category of posttranslational modification, protein turnover, and chaperones. The expression levels of 8 proteins were increased, whereas those of 6 proteins were decreased after GZZSZTW treatment. Among the upregulated proteins, Pzp, Ovos, and Cast were proteinase inhibitors that cripple the proteases and prevent the degradation of proteins. Pzp and Ovos are proteinase inhibitors that are able to inhibit all four classes of proteinases [29, 30]. Cast is a specific, suicide inhibitor of calpain, which is a calcium-dependent cysteine protease [31]. These results suggest that GZZSZTW treatment confers the primary chondrocyte the capacity to prevent protein degradation, which is consistent with the effects of GZZSZTW for treating OA. In addition to Marcksl1 and Sncg, which were involved in cytoskeleton regulation, the expression level of another protein, namely, matrix Gla protein (Mgp), was also significantly increased after GZZSZTW treatment. Mgp is a powerful but developmentally regulated inhibitor of cartilage mineralization. Constitutive Mgp expression in the limb not only inhibits cartilage mineralization but also blocks chondrocyte maturation and intramembranous and endochondral ossification [32]. Therefore, the increased expression level of Mgp upon GZZSZTW treatment plays a key role in the prevention of primary chondrocyte maturation and mineralization, which could be an underlying mechanism for OA treatment with GZZSZTW. The expression levels of several differentially expressed proteins were significantly decreased upon GZZSZTW treatment, including Afp, Ltf, Alb, Ctrb1, Ahsg, and Klk1b1. Among these proteins, Ltf plays a negative role in regulating chondrocyte homeostasis by inhibiting aggrecan synthesis and increasing the levels of catabolic indicators in chondrocytes [33]. Ahsg, also known as fetuin-A, plays a role in inducing the expression of alkaline phosphatase during chondrocyte differentiation [34]. Ctrb1 and Klk1b are proteinases that perform proteolysis to break down proteins and polypeptides. Ctrb1 is the precursor of the proteolytic enzyme chymotrypsin, whereas Klk1b1 is a member of the kallikrein subfamily, which show trypsin- or chymotrypsin-like serine proteases properties [35, 36]. These results suggest that GZZSZTW treatment significantly decreases the expression of proteinases and prevents the breakdown of the extracellular matrix of chondrocytes. Our results also suggest that GZZSZTW treatment may prevent chondrocytes differentiation.

According to the eggNOG annotation, there were 11 differentially expressed proteins that were classified into the category of signal transduction mechanisms, and the levels of almost all of these proteins were increased subsequent to GZZSZTW treatment. In addition to the proteins that we have previously discussed, including Krt79, Marcksl1, and Ahsg, several other proteins that might play key roles in chondrocytes were identified: Csf3r, Dap, Phlpp2, Sh3glb1, Pth2, Cfdp1, Akap8, and Dab2. Among these proteins, Csf3r is a cell surface receptor for the granulocyte colony-stimulating factor, which plays a pivotal role during the process of cartilage repair [37]. Dap, Phlpp2, and Sh3glb1 are important mediators involved in cell survival and death through regulating multiple signal transduction pathways [38–40]. Pth2, a ligand for the parathyroid hormone 2 receptor, plays a pivotal role in regulating chondrocyte proliferation and differentiation [41]. Akap8 is a member of the A-kinase anchoring protein family that is mainly expressed in the matrix of precartilage but not in fully differentiated cartilage [42]. These results suggest that GZZSZTW regulates chondrocyte survival and death through multiple signal transduction mediators.

According to the eggNOG annotation, there were 11 differentially expressed proteins that were classified into the category of chromatin structure and dynamics, and the levels of almost all of these proteins were increased after GZZSZTW treatment. Among these proteins, Ptms, a small nuclear protein, interacts with the H1 linker histones (e.g., Hist1h1b, Hist1h1d, and Hist1h1e), which are synthesized during DNA synthesis and are associated with cell proliferation, chromatin remodeling, and gene transcription [43–45]. Mecp2 is a nuclear protein that maintains DNA methylation by regulating multiple regulatory complexes during DNA replication [46]. Hmga1 is not only a nuclear protein that is associated with chromatin remodeling but also an important protein for mitochondrial DNA maintenance and organelle function [47]. Gapdh was initially considered as a housekeeping gene. However, Gapdh may be highly upregulated in some cancers that are correlated with excessive cell proliferation [48]. Tdrd1 is a direct gene target of the transcription factor Erg that is strongly associated with primary prostate cancer [49]. These results suggest that GZZSZTW treatment increases the expression levels of proteins that regulate chromatin structure and dynamics, which consequently accelerate chondrocyte proliferation.

According to the eggNOG annotation, there were 10 differentially expressed proteins that were classified into the category of translation, ribosomal structure, and biogenesis, and the expression of almost all these proteins was increased after GZZSZTW treatment. The majority of these proteins belonged to the 40S and 60S ribosomal protein subunits including Rps19, Rps27l, Rpl34, Rpl37, Rps28, Rps23, Rpl35, and Rpl36. These proteins not only participate in translation, ribosomal structure, and biogenesis but also play a crucial role in the regulation of cell proliferation. For instance, an Rps19 deficiency in hematopoietic cells significantly decreased cell proliferation rates, and then Rps19 overexpression in these cells improved or even rescued this event [50]. Overexpression of Rpl34 promotes cell proliferation and is involved in many types of cancer, including human non-small cell lung cancer, gastric cancer, and pancreatic cancer [51]. Furthermore, RPL36 promotes cell proliferation and G1/S cell cycle progression in glioma [52]. These results suggest that GZZSZTW treatment significantly accelerates the process of translation and aids ribosomal structure and biogenesis by increasing expression levels of the 40S and 60S ribosomal proteins, and these upregulated proteins, in turn, promote chondrocyte proliferation.

## 5. Conclusion

The present study demonstrates that the Chinese medicinal formulation GZZSZTW, which has been widely used to treat joint diseases, modulates chondrocyte structure, dynamics, and metabolism. Those were achieved by controlling multiple cellular processes, such as DNA replication and transcription, protein synthesis and degradation, cytoskeleton dynamics, and signal transduction in chondrocytes. GZZSZTW treatment significantly increased the levels of cytoskeletal proteins, proteinase inhibitors, signaling regulators, and nuclear proteins. However, GZZSZTW treatment significantly decreased the expression levels of proteinases that are capable of hydrolyzing proteins. Furthermore, GZZSZTW treatment significantly increased the levels of proteins that play key roles in promoting chondrocyte proliferation and prevent chondrocyte differentiation and mineralization. Thus, this study has contributed to the current knowledge of the molecular mechanism by which GZZSZTW acts on chondrocytes. It has also shed light on possible strategies to further prevent and treat cartilage-related diseases by using traditional Chinese medicinal formulations.

## Figures and Tables

**Table 1:** KEGG enrichment analysis of differentially expressed proteins (GZZSZTW versus Blank).

Pathways	Number of genes
Translation	12
Transport and catabolism	6
Signal transduction	6
Immune system	5
Infectious diseases: viral	4
Cell growth and death	3
Cancers: overview	3
Endocrine system	3
Cellular community - eukaryotes	2
Transcription	2
Cardiovascular diseases	2
Infectious diseases: bacterial	2
Neurodegenerative diseases	2
Carbohydrate metabolism	2
Global and overview maps	2
Digestive system	2
Cell motility	1
Membrane transport	1
Signaling molecules and interaction	1
Drug resistance: antineoplastic	1
Immune diseases	1
Infectious diseases: parasitic	1
Substance dependence	1
Excretory system	1
Sensory system	1

**Table 2:** Subcellular distribution of differentially expressed proteins (GZZSZTW versus Blank).

Subcellular location	Number of genes
Nucleus	35
Cytoplasm	18
Extracellular space	14
Mitochondria	6
Plasma membrane	6
Cytoplasm & nucleus	3
Cytoskeleton	2

**Table 3:** Statistics of eggNOG classification of differentially expressed proteins (GZZSZTW versus Blank).

Categories	Number of genes
Cytoskeleton	17
Posttranslational modification, protein turnover, chaperones	14
Signal transduction mechanisms	11
Chromatin structure and dynamics	11
Translation, ribosomal structure and biogenesis	10
Transcription	8
Extracellular structures	7
Intracellular trafficking, secretion, and vesicular transport	5
RNA processing and modification	2
Amino acid transport and metabolism	2
Inorganic ion transport and metabolism	2
Carbohydrate transport and metabolism	1
Replication, recombination and repair	1
Cell cycle control, cell division, chromosome partitioning	1
Energy production and conversion	1

**Table 4:** Differentially expressed proteins that were classified into cytoskeleton (GZZSZTW versus Blank).

Protein name	Fold change	p value
Keratin, type II cytoskeletal 79 (Krt79)	2.660	0.001
Keratin, type I cytoskeletal 17 (Krt17)	2.446	0.044
Tubulin alpha-1B chain (Tuba1b)	2.309	0.015
Keratin, type I cytoskeletal 15 (Krt15)	2.187	0.022
Keratin, type I cytoskeletal 42 (Krt42)	2.112	0.001
Keratin, type I cytoskeletal 16 (Krt16)	2.027	0.001
Keratin, type I cytoskeletal 10 (Krt10)	2.023	0.001
MARCKS-related protein (Marcksl1)	1.993	0.012
Keratin, type II cytoskeletal 5 (Krt5)	1.973	0.001
Keratin, type II cytoskeletal 6A (Krt6a)	1.908	0.001
Keratin, type I cytoskeletal 14 (Krt14)	1.727	0.003
Keratin, type II cytoskeletal 1 (Krt1)	1.708	0.001
Desmoplakin (Dsp)	1.661	0.001
Septin-7 (Sept7)	1.623	0.001
Keratin, type II cytoskeletal 2 (Krt2)	1.575	0.004
Tropomodulin-2 (Tmod2)	1.558	0.001
Gamma-synuclein (Sncg)	1.521	0.011

**Table 5:** Differentially expressed proteins that were classified into posttranslational modification, protein turnover, chaperones (GZZSZTW versus Blank).

Protein name	Fold change	Q value
Pregnancy zone protein (Pzp)	2.088	0.001
MARCKS-related protein (Marcksl1)	1.993	0.012
Ovostatin homolog (Ovos)	1.955	0.007
Tuberoinfundibular peptide of 39 residues (Pth2)	1.688	0.003
Calpastatin (Cast)	1.631	0.001
UBX domain-containing protein 4 (Ubxn4)	1.580	0.001
Matrix Gla protein (Mgp)	1.535	0.003
Gamma-synuclein (Sncg)	1.521	0.011
Alpha-fetoprotein (Afp)	0.637	0.001
Lactotransferrin (Ltf)	0.550	0.003
Serum albumin (Alb)	0.545	0.001
Chymotrypsinogen B (Ctrb1)	0.511	0.001
Alpha-2-HS-glycoprotein (Ahsg)	0.364	0.001
Kallikrein 1-related peptidase b1 (Klk1b1)	0.291	0.019

**Table 6:** Differentially expressed proteins that were classified into signal transduction mechanisms (GZZSZTW versus Blank).

Protein name	Fold change	Q value
Granulocyte colony-stimulating factor receptor (Csf3r)	8.552	0.001
Death-associated protein 1 (Dap)	3.140	0.001
Keratin, type II cytoskeletal 79 (Krt79)	2.660	0.001
MARCKS-related protein (Marcksl1)	1.993	0.012
PH domain leucine-rich repeat-containing protein phosphatase 2 (Phlpp2)	1.935	0.003
Endophilin-B1 (Sh3glb1)	1.724	0.001
Tuberoinfundibular peptide of 39 residues (Pth2)	1.688	0.003
Craniofacial development protein 1 (Cfdp1)	1.683	0.015
A-kinase anchor protein 8(Akap8)	1.508	0.008
Disabled homolog 2 (Dab2)	1.500	0.001
Alpha-2-HS-glycoprotein (Ahsg)	0.364	0.001

**Table 7:** Differentially expressed proteins that were classified into chromatin structure and dynamics (GZZSZTW versus Blank).

Protein name	Fold change	Q value
Parathymosin (Ptms)	3.085	0.001
Histone H1.5 (Hist1h1b)	2.104	0.001
Methyl-CpG-binding protein 2 (Mecp2)	2.056	0.040
High mobility group protein HMG-I/HMG-Y (Hmga1)	1.959	0.001
Histone H1.3 (Hist1h1d)	1.950	0.005
Histone H1.4 (Hist1h1e)	1.901	0.001
Glyceraldehyde-3-phosphate dehydrogenase (Gapdh)	1.547	0.001
Histone H1.1 (Hist1h1a)	1.530	0.001
Histone H2A.V (H2afv)	1.515	0.007
A-kinase anchor protein 8 (Akap8)	1.508	0.008
Tudor domain-containing protein 1 (Tdrd1)	0.619	0.001

**Table 8:** Differentially expressed proteins that were classified into translation, ribosomal structure, and biogenesis (GZZSZTW versus Blank).

Protein name	Fold change	Q value
40S ribosomal protein S19 (Rps19)	1.843	0.001
40S ribosomal protein S27-like (Rps27l)	1.713	0.026
60S ribosomal protein L34 (Rpl34)	1.645	0.001
60S ribosomal protein L37 (Rpl37)	1.582	0.008
40S ribosomal protein S28 (Rps28)	1.580	0.001
40S ribosomal protein S23 (Rps23)	1.546	0.001
60S ribosomal protein L35 (Rpl35)	1.537	0.001
Eukaryotic translation initiation factor 1A, X-chromosomal (Eif1ax)	1.512	0.001
60S ribosomal protein L36 (Rpl36)	1.508	0.037
60S acidic ribosomal protein P1 (Rplp1)	0.332	0.001

**Table 9:** Differentially expressed proteins that were classified into other eggNOG categories (GZZSZTW versus Blank).

Protein name	Fold change	Q value
*Transcription*		
Death-associated protein 1 (Dap)	3.140	0.001
Protein S100-A1 (S100a1)	2.538	0.009
Methyl-CpG-binding protein 2 (Mecp2)	2.056	0.040
High mobility group protein HMG-I/HMG-Y (Hmga1)	1.959	0.001
Prothymosin alpha (Ptma)	1.890	0.016
Nuclear factor 1 A-type (Nfia)	1.787	0.028
Tudor domain-containing protein 1 (Tdrd1)	0.619	0.001
Lactotransferrin (Ltf)	0.550	0.003
*Extracellular structures*		
Granulocyte colony-stimulating factor receptor (Csf3r)	8.552	0.001
Collagen alpha-1(X) chain (Col10a1)	2.161	0.001
Matrix Gla protein (Mgp)	1.535	0.003
Alpha-fetoprotein (Afp)	0.637	0.001
Lactotransferrin (Ltf)	0.550	0.003
Serum albumin (Alb)	0.545	0.001
Alpha-2-HS-glycoprotein (Ahsg)	0.364	0.001
*Intracellular trafficking, secretion, and vesicular transport*		
Granulocyte colony-stimulating factor receptor (Csf3r)	8.552	0.001
Sorting nexin-3 (Snx3)	1.865	0.027
Ran-specific GTPase-activating protein (Ranbp1)	1.641	0.001
Mitochondrial fission factor (Mff)	1.503	0.010
Disabled homolog 2 (Dab2)	1.500	0.001
*RNA processing and modification*		
Prothymosin alpha (Ptma)	1.890	0.016
Serine/arginine-rich splicing factor 5 (Srsf5)	1.526	0.001
*Amino acid transport and metabolism*		
Chymotrypsinogen B (Ctrb1)	0.511	0.001
Kallikrein 1-related peptidase b1 (Klk1b1)	0.291	0.019
*Inorganic ion transport and metabolism*		
Lactotransferrin (Ltf)	0.550	0.003
Zinc transporter ZIP8 (Slc39a8)	0.395	0.001
*Carbohydrate transport and metabolism*		
Glyceraldehyde-3-phosphate dehydrogenase (Gapdh)	1.547	0.001
Replication, recombination and repair		
Prothymosin alpha (Ptma)	1.890	0.016
*Cell cycle control, cell division, chromosome partitioning*		
Septin-7 (Sept7)	1.623	0.001
*Energy production and conversion*		
Prothymosin alpha (Ptma)	1.890	0.016

**Table 10:** Validation of differentially expressed proteins using PRM assay (GZZSZTW versus Blank).

Protein name	Fold change	p value	Fold change	p value
	(iTRAQ)	(iTRAQ)	(PRM)	(PRM)
Keratin, type I cytoskeletal 42 (Krt42)	2.112	0.001	1.946	0.005
Keratin, type II cytoskeletal 5 (Krt5)	1.973	0.001	1.283	0.022
Keratin, type I cytoskeletal 16 (Krt16)	2.027	0.001	1.170	0.000
Matrix Gla protein (Mgp)	1.535	0.003	3.538	0.002
Pregnancy zone protein (Pzp)	2.088	0.001	1.733	0.000
60S ribosomal protein L35 (Rpl35)	1.537	0.001	1.923	0.021
40S ribosomal protein S23 (Rps23)	1.546	0.001	1.796	0.031
Craniofacial development protein 1 (Cfdp1)	1.683	0.015	1.847	0.030
Parathymosin (Ptms)	3.085	0.001	2.421	0.008
Histone H1.5 (Hist1h1b)	2.104	0.001	1.924	0.013
Methyl-CpG-binding protein 2 (Mecp2)	2.056	0.040	1.044	0.004
Histone H1.1 (Hist1h1a)	1.530	0.001	1.713	0.030

## Data Availability

The data used to support the findings of this study are available from the corresponding author upon request.
